# The Effects of 17-Methoxyl-7-Hydroxy-Benzene-Furanchalcone on the Pressure Overload-Induced Progression of Cardiac Hypertrophy to Cardiac Failure

**DOI:** 10.1371/journal.pone.0091834

**Published:** 2014-03-12

**Authors:** Jianchun Huang, XiaoJun Tang, Xingmei Liang, Qingwei Wen, Shijun Zhang, Feifei Xuan, Jie Jian, Xing Lin, Renbin Huang

**Affiliations:** 1 Pharmaceutical College, Guangxi Medical University, Nanning, Guangxi, China; 2 Department of Laboratory Medicine, Guangxi Medical College, Nanning, Guangxi, China; Universidade Federal do Rio de Janeiro, Brazil

## Abstract

We investigated the effects of 17-methoxyl-7-hydroxy-benzene-furanchalcone (MHBFC), which was isolated from the roots of *Millettia pulchra* (Benth.) Kurz var. *Laxior* (Dunn) Z.Wei (Papilionaceae) (MKL), on the progression of cardiac hypertrophy to failure in a rat model of abdominal aortic banding (AAB)-induced pressure overloading. Endothelial dysfunction is central to pressure overload-induced cardiac hypertrophy and failure. It would be useful to clarify whether MHBFC could prevent this dysfunction. The effects of pressure overload were assessed in male Sprague–Dawley rats 6 weeks after AAB using the progression of cardiac hypertrophy to heart failure as the endpoint. The AAB-treated rats exhibited a greater progression to heart failure and had significantly elevated blood pressure, systolic and diastolic cardiac dysfunction, and evidence of left ventricular hypertrophy (LVH). LVH was characterized by increases in the ratios of heart and left ventricular weights to body weight, increased myocyte cross-sectional areas, myocardial and perivascular fibrosis, and elevated cardiac hydroxyproline. These symptoms could be prevented by treatment with MHBFC at daily oral doses of 6 and 12 mg/kg for 6 weeks. The progression to cardiac failure, which was demonstrated by increases in relative lung and right ventricular weights, cardiac function disorders and overexpression of atrial natriuretic peptide (ANP) mRNA, could also be prevented. Furthermore, MHBFC partialy rescued the downregulated nitric oxide signaling system, whereas inhibited the upregulated endothelin signaling system, normalizing the balance between these two systems. MHBFC protected the endothelium and prevented the pressure overload-induced progression of cardiac hypertrophy to cardiac failure.

## Introduction

Hypertension is a continuum that starts with a rise in blood pressure, evolves to left ventricular hypertrophy (LVH), proteinuria or endothelial dysfunction, and, insofar as it is not adequately treated or controlled, finally leads to the development of complications, the most relevant of which are stroke and heart failure [1]. Hypertensive disease is the most frequent background of LVH, and it is generally felt that anti-hypertensive treatment should not only lower blood pressure but also cause the regression of LVH [2]. Various noxious sequelae of cardiovascular diseases and conditions, such as coronary heart disease, stroke, congestive heart failure, and sudden death, are known to be aggravated by LVH [3]. Endothelial dysfunction is known to play important roles in the pathogenesis and progressiveness of hypertensive heart disease [4], [5]. The pathophysiological mechanisms of endothelial dysfunction that are related to a decrease in the bioavailability of NO as well as to augmented ET-1 synthesis, release, or activity [6]. Thus, a subsequent decrease in NO bioavailability is fundamental to the LVH process. In hypertension, reduction of BP *per se* does not seem to restore endothelial function. Angiotensin receptor blockers and angiotensin-converting enzyme inhibitors have been shown to be especially beneficial [7]–[9]. There is current evidence demonstrating that the best drugs that achieve these goals are renin-angiotensin system blockers (ACEI or angiotensin-receptor blocking agents) and calcium channel blockers, as evidenced by three large trials, LIFE ASCOT and ACCOMPLIISH, with more than 40000 patients [1]. In animal models of hypertension, oxidative excess leads to endothelial dysfunction as evidenced by improvement of the impaired endothelium-dependent relaxation after use of antioxidants [10]. Oxidative excess in hypertensive patients leads to diminished NO [11] and correlates with the degree of impairment of endothelium-dependent vasodilation and with cardiovascular events [12]. Recently, increased attention has been focused on traditional Chinese herbal treatments because of their unique decrease in oxidative stress efficacy and little adverse reactions.

*Millettia pulchra* (Benth.) Kurz var. *Laxior* (Dunn) Z.Wei (Papilionaceae) (MKL) is a traditional Chinese medicinal herb that is extensively distributed in the Guangxi Province of China. Our previous studies have demonstrated that extracts of MKL roots have antihypertensive, antioxidative, anti-inflammatory effects [13]–[16]. Additionally, the drugs of these previous studies were the total extracts of MKL roots, and MHBFC is a flavonoid monomer that was originally isolated from a 60% ethanol extract from MKL roots [17]. Previous studies have demonstrated that MHBFC could scavenge hydroxyl radicals and oxyradicals [18], enhance the cardiocyte survival rate in hypoxia/reoxygenation injury [19], and protect the heart against myocardial ischemia *in vitro* and *in vivo* [20]. Based on the above information, we hypothesized that MHBFC might be effective in the treatment of hypertensive heart disease. Here, for the first time, we investigated its effects on cardiovascular remodeling that is induced by a pressure overload as well as the potential mechanisms that are involved.

The abdominal aortic stenosis model using rats is a pressure-overload model that is similar to the progression of hypertensive heart disease. In this condition, LVH with myocyte hypertrophy and interstitial fibrosis develops in response to a sustained elevation of LV systolic pressure in the presence of systemic hypertension 6 weeks after abdominal aortic banding (AAB) [21], [22]. Here, we investigated the effects of MHBFC on cardiac hypertrophy and cardiac failure that was induced by pressure overloading using AAB in rats, exploring the potential mechanisms that were involved in endothelial protection. From our data, we suggest that MHBFC might be effective in the treatment of hypertensive heart disease via the molecular pathways that are involved in endothelial protection.

## Materials and Methods

### 2.1: Chemicals

MHBFC (purity >95%) was isolated from MKL by the Department of Pharmacology, Guangxi Medical University (Guangxi, China), characterized by UV, IR, ESI-MS, NMR, and X-ray monocrystal diffraction [17], [20]. NG-nitro-L-arginine methyl ester (L-NAME) was purchased from Shanghai Yuanye Bio-Technology Co., Ltd. All other chemicals and materials were obtained from local commercial sources.

### 2.2: Abdominal Aortic Banding Model and Protocol

Male Sprague–Dawley rats with a body weight of 130–160 g were obtained from the Guangxi Medical Laboratory Animal Center. Rats were housed under standard conditions (temperature at 20–25°C, relative humidity at 50–60%). The animal experiments were approved by the Animal Ethics Committee of the Guangxi Medical University and performed in accordance with their guidelines. After surgery, the rats were divided randomly into 7 groups (n = 6): sham-operated group, animals underwent a similar procedure without banding the aorta; model group, pressure-overloaded rats induced by AAB above the renal arteries and treated with distilled water; AAB rats treated with MHBFC 6 or 12 mg kg^−1^day^−1^; AAB rats treated with lisinopril, an angiotensin-converting enzyme I (ACEI), 15 mg kg^−1^day^−1^ (the effects of lisinopril on the LVH that was induced by pressure overload has been well documented. In this study, lisinopril was used as a positive control drug to demonstrate the reliability of AAB rats under our experimental conditions); AAB rats treated with L-NAME 50 mg kg^−1^day^−1^; and AAB rats treated with MHBFC 12 mg kg^−1^day^−1^ plus L-NAME 50 mg kg^−1^day^−1^. No significant difference was found among all of the experimental rats in age and body weight before surgery, using a pressure-overload model as described previously with minor modifications [22]. Briefly, the rats were anaesthetized with a 10% chloral hydrate (3 ml kg^−1^, intraperitoneal injection). Under sterile conditions, the abdominal aorta above the kidneys was exposed through a midline abdominal incision and constricted at the suprarenal level by a 4-0 silk suture tied around both the aorta and a blunted 22 gauge needle, which was then pulled out. A similar procedure was performed for Sham, without the ligature. The drugs were dissolved in distilled water and administered orally via a gastric tube. MHBFC or vehicle was orally administered once a day in 2 mL kg^−1^ for 6 weeks after surgery. At the last day of 0, 2, 4 and 6 weeks, the relevant transducer was connected to a MS 4000 biological signal quantitative analytical system (Longfeida Technology Co., Ltd.), conscious systolic blood pressure (SBP) and heart rates (HRs) were monitored by the tail-cuff method as we described previously [20], and the researchers blinded to the identity of the rats during the recordings. No significant difference was found among all of the experimental groups in body weight, SBP and HRs at 0 week.

### 2.3: Hemodynamics and Cardiac Remodeling Index

On day 42, all animals were anesthetized with 10% chloral hydrate (3 ml kg^−1^, intraperitoneal injection), the right carotid arteries were cannulated with a polyethylene catheter that was connected to a Statham transducer, and the mean carotid pressures were measured.

Then, the polyethylene cannula was inserted along the right coronary artery into the left ventricle, and electrodes were plugged in the limbs for electrocardiography. The relevant transducer was connected to an MS 4000 organism signal quantitative analytical system (Longfeida Technology Co., Ltd.), and the hemodynamic parameters (including HR, SP, DP, AP, LVSP, LVEDP, +dp/dtmax, and −dp/dtmax) were recorded.

Thereafter, blood samples were collected and animals were killed by exsanguination. The thoracic cavity was opened to expose the still-beating heart. The hearts and lungs were rapidly removed, rinsed in ice-cold 0.9% NaCl solution, blotted, and weighed. The heart-weight index (HW/BW) was calculated by dividing heart weight by body weight, and then the left ventricles (including interventricular septum) and right ventricular free walls were collected separately and weighed. The left ventricular-weight index (LVW/BW), right ventricular-weight index (RVW/BW), and lung index (LW/BW) were then calculated.

### 2.4: Histological Analysis

The hearts were immersion-fixed in neutral 10% buffered formalin, and paraffin sections (5 mm) were cut. The myocyte cross-sectional area and myocardial fibrosis were quantitatively analyzed with the Image J2× software. For the measurement of the cross-sectional area, 50 cells (per animal, in hematoxylin and eosin stain, ×400) from the left ventricular lateral-mid free wall (including epicardial and endocardial portions) were randomly selected and analyzed. Myocardial fibrosis in the tissue sections was quantitatively analyzed by morphometry in two ways (in Masson’s trichrome stained sections, ×100): (i) The perivascular fibrosis of arteries was evaluated in short-axis images of intramuscular arteries and arterioles (at least 10 per animal). The area occupied by the artery (A) and the area of fibrosis surrounding the artery (B) were traced and calculated. The perivascular fibrosis index was defined as B/A. (ii) The collagen in myocardial interstitial spaces, excluding perivascular areas, was visualized, and the whole areas of the sections were scanned. The total interstitial fibrosis index was defined as the sum of the total area of collagen in the entire visual field, divided by the sum of total connective tissue area, plus the myocardial area in the entire visual field. All of the images were digitalized and transformed into binary images, and the areas occupied by collagen were calculated by an automatic area-quantification program in the Image J2× software.

After treatment for 6 weeks, the animals were killed, the sections at the same spot along the aorta were obtained and the structural changes of aorta were investigated using a light microscope. The total aortic area (TAA), lumen area (LA), cross-sectional area (CSA), aorta diameter (AD), luminal radius (L), and media thickness (M) of aorta were recorded under a light microscope, and the ratio of M/L was calculated.

### 2.5: Nitric Oxide, Endothelin-1, and Hydroxyproline Measurement

Within 30 s after collection, heparinized blood was centrifuged for 10 min at 3000 rpm, and all samples were stored at −80°C until assayed. Because of its instability in physiological solutions, most of the NO is rapidly converted to nitrite (NO_2_^−^) and further to nitrate (NO_3_^−^). Plasma levels of NO_2_^−^/NO_3_^−^ were measured using a NO detection kit according to the manufacturer’s instructions. Briefly, nitrate was converted to nitrite using aspergillus nitrite reductase, and the total nitrite was measured with the Griess reagent. The absorbance was determined at 540 nm with a spectrophotometer. ET-1 was measured using a rat Endothelin 1 Elisa kit (CUSABIO BIOTECH Co., Ltd.) according to the manufacturer’s instructions. The contents of hydroxyproline in cardiac muscle were measured using a commercial kit (Nanjing Jiancheng Bioengineering Institute, Nanjing, China) according to the manufacturer’s instructions.

### 2.6: Immunohistochemistry of Endothelial Nitric Oxide Synthase, Endothelin Receptor A, and Endothelin Receptor B Expression

Immunohistochemical staining was performed using an UltraSensitive TM S-P kit according to the manufacturer’s instructions. Briefly, the sections were deparaffinized and microwave-treated for 10 min twice in 10 mM sodium citrate (pH 6.0). Endogenous peroxidase was blocked by incubating the sections in endogenous peroxidase blocking solution for 10 min at room temperature. Rabbit polyclonal antibodies against endothelial NO synthase (eNOS), endothelin receptor A (ET_A_), and endothelin receptor B (ET_B_) proteins were used as primary antibodies in a 1∶100 dilution at 4°C for 18 h. After washing three times with phosphate-buffered saline (PBS), sections were incubated with biotin-conjugated anti-rabbit secondary antibody for 10 min. The sections were then washed three times with PBS, treated with streptavidin-peroxidase for 10 min, and then washed again with PBS three times. Lastly, the specimens were incubated in diaminobenzidine for 5 min, followed by hematoxylin counterstaining. Images from all sections were acquired using a digital camera system. Confocal images were then transferred to a personal computer using the image analysis software package Image J2×.

### 2.7: Reverse Transcription-polymerase Chain Reaction

Total RNA was extracted from the LV tissue of rats using TRIzol reagent according to the manufacturer’s instructions. The total RNA was then converted to cDNA using reverse transcriptase with random hexamer priming. PCR was performed by standard methods, as described in a previous report [22], using synthetic gene-specific primers for ANP, ET-1, endothelin-converting enzyme (ECE), and β-Actin (Table 1). The parallel amplification of rat β-Actin was performed for reference, and the intensity of each band was quantified using densitometry. The intensity of each gene band was expressed relative to the corresponding densities of the β-Actin bands from the same RNA samples.

**Table 1:** Oligonucleotide primers used for reverse transcription-polymerase chain reaction.

Target gene	Accession no.	Primers (5′–3′)	Cycle program T (°C)	Length(bp)
ANP	NM-012612	F: GGC TCC TTC TCC ATC ACC AA	52	268
		R: TCT GAG ACG GGT TGA CTT CC		
ET-1	NM-012548	F: TGG CTT TCC AAG GAG CTC	58	394
		R: GCT TGG CAG AAA TTC CAG		
ECE	NM-053596	F: TGA GCA CCC TGA AAT GGA	56	488
		R: CTG CTG CTT GAA TGC CTC		
β-Actin	NM_031144.3	F: AGG CAT CCT GAC CCT GAA GTA C	60	389
		R: TCT TCA TGA GGT AGT CTG TCA G		
β-Actin	NM_031144.3	F: AAC CCT AAG GCC AAC CGT GAA AAG	60	240
		R: TCA TGA GGT GGT AGT CTG TCA GGT		

### 2.8: Statistical Analysis

The results were presented as the means ± SD, and a statistical analysis was performed with the Sigma Stat (version 13.0) statistical program (SPSS Inc., Chicago, IL, USA). Differences between groups were tested for statistical significance using a one-way analysis of variance (ANOVA) performed with S-N-K post-test. Differences were considered statistically significant at *P*-values that were less than 0.05.

## Results

### 3.1: Hemodynamic Effects of MHBFC

After applying AAB above the renal arteries, the HR and SBP didn’t show significant difference between the groups at 0 week but increased significantly at 2, 4 and 6 weeks compared with sham-operated rats. MHBFC, at the daily oral doses of 6 and 12 mg kg^−1^ for 6 weeks, prevented increases in HR and SBP (Table 2). The carotid arterial pressure and cardiac functions were measured 6 weeks after AAB. As shown in Table 3, compared with those measurements in sham-operated rats, right carotid aorta systolic blood pressure (ASBP), diastolic blood pressure (ADBP) and aorta mean blood pressure (AMBP) were significantly elevated in AAB-treated rats. These features were prevented by treatment with MHBFC at all doses for 6 weeks. The measurements of the in vivo function of LV for all groups are shown in Table 4. Systolic cardiac parameters, including LVSP, +d*p*/d*t*_max_ and diastolic cardiac parameter −d*p*/d*t*_max_, were all significantly elevated in AAB-treated rats. In contrast, LVEDP decreased significantly. These changes could also be prevented by treatment with MHBFC.

**Table 2:** Effect of MHBFC on heart rate (HR) and systolic blood pressure (SBP) in different groups during the course of the experiments (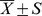, n = 6).

Group	0 week	2 week	4 week	6 week
**Heart rate (b.p.m.)**				
Sham	359±37	361±38	358±21	360±23
Model	361±33	380±25	388±23#	389±21#
MHBFC 6 mg/kg	365±32	372±20	372±20	371±26
MHBFC 12 mg/kg	356±29	373±26	369±29	365±29
Lisenopril 15 mg/kg	364±36	373±41	368±39	365±41
**Systolic blood pressure (tail-cuff) (mmHg)**				
Sham	105.53±8.44	104.87±8.21	106.09±6.97	105.87±7.92
Model	103.80±4.73	116.40±5.13#	118.31±4.08##	121.64±4.50##
MHBFC 6 mg/kg	104.19±4.93	113.18±4.17	111.20±3.94*	110.02±3.62**
MHBFC 12 mg/kg	104.55±5.74	110.55±6.97	107.97±7.32*	107.08±8.31**
Lisenopril 15 mg/kg	104.30±6.38	108.72±4.38*	106.20±3.67**	105.09±4.00**

# P<0.05,

## P<0.01 vs Sham group;

*P<0.05,

**P<0.01 vs Model group.

**Table 3:** Effects of MHBFC on carotid ASBP, ADBP, AMBP and body weight in pressure-overload rats (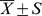, n = 6).

Group	ASBP (kPa)	ADBP (kPa)	AMBP (kPa)	body weight (g)
Sham	16.38±1.06	12.62±1.44	13.53±1.56	303.75±8.87
Model	24.58±2.44##	16.75±1.90##	19.20±1.72##	296.18±6.79
MHBFC 6 mg/kg	22.23±1.06	14.40±1.46*	16.57±1.05**	297.81±6.57
MHBFC 12 mg/kg	19.43±2.57 ** $	13.53±1.52**	15.85±1.27**	296.20±7.93
Lisenopril 15 mg/kg	19.78±2.37 **	13.30±1.85**	15.35±2.37**	300.54±10.42

ASBP: aorta systolic blood pressure; ADBP: aorta diastolic blood pressure; AMBP: aortamean blood pressure.

## P<0.01 vs Sham group;

*P<0.05,

**P<0.01 vs Model group;

$ P<0.05 vs MHBFC 6 mg/kg.

**Table 4:** Effects of MHBFC on left ventricular function in pressure-overload rats (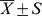, n = 6).

Group	HR(beats/min)	LVEDP(kPa)	LVSP(kPa)	+dp/dt_max_(kPa/s)	−dp/dtmax(kPa/s)
Sham	382.3±32.0	4.02±1.24	15.90±1.99	432.00±44.96	404.62±38.46
Model	411.7±33.2	1.18±0.97##	19.72±1.75##	533.95±35.82##	482.58±29.57##
MHBFC 6 mg/kg	379.0±33.4	2.07±0.97	17.45±1.56*	492.22±27.13*	451.12±30.68
MHBFC 12 mg/kg	362.8±40.9*	3.28±1.14**	16.25±1.04**	466.47±47.04*	421.20±28.83**
Lisenopril 15 mg/kg	366.7±32.1*	3.05±1.03**	15.85±1.94**	464.85±38.06**	419.80±19.97**

HR: heart rate; LVSP: left ventricular systolic pressure; LVEDP: left ventricular end-diastolic pressure; +dp/dtmax: maximal rate of left ventricular systolic pressure; −dp/dtmax: maximal rate of left ventricular diastolic pressure.

## P<0.01 vs Sham group;

*P<0.05,

**P<0.01 vs Model group.

### 3.2: MHBFC Reverses the Aortic Remodeling that was Induced by a Pressure Overload

**Figure 1:**
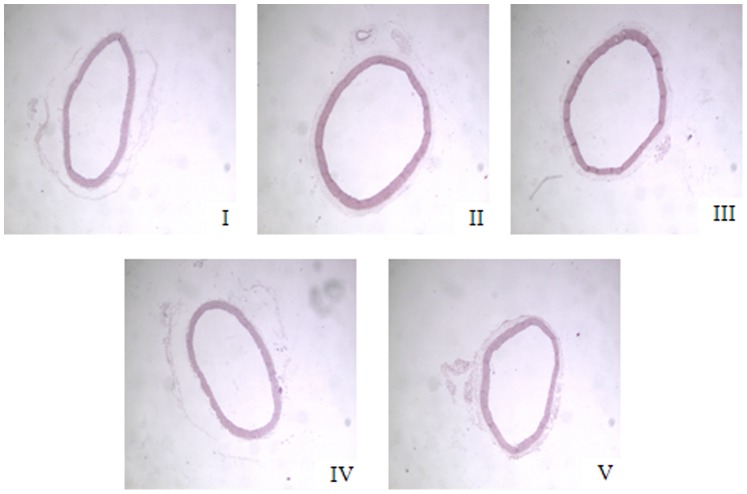
Representative figure of aorta remodeling in different groups. I: Sham group; II: Model group; III: MHBFC 6 mg kg^−1^ group; IV: MHBFC 12 mg kg^−1^ group; V: Lisinopril 15 mg kg^−1^ group. The hypertensive vascular remodelling was observed by increases in the area of the total aorta, aorta lumen and aorta cross-sectional, which could be prevented by treatment with MHBFC at all doses for 6 weeks.

The hypertensive vascular remodeling of the upper thoracic aorta that was exposed to a pressure overload by narrowing the abdominal aorta was observed 6 weeks after AAB. The values of the area of the TAA, LA, CSA, AR, Lumen, Media and M/L ratio of the aorta in AAB-treated rats increased significantly compared with sham-operated rats. These changes could be prevented by treatment with MHBFC at all doses for 6 weeks (Table 5, Figure 1).

**Table 5:** Effect of MHBFC on aorta remodeling in pressure-overload rats. (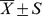, n = 6).

Group	TAA/×10^3^ µm^2^	LA/×10^3^ µm^2^	CSA/×10^3^ µm^2^	CSA/TAA/%	AD/µm	Lumen/µm	Media/µm	Media/Lumen/%
Sham	4135.26±388.12	2794.80±253.49	1340.47±275.61	32.26±4.42	2293.01±109.16	1885.26±85.23	203.88±37.95	10.84±2.12
Model	6115.76±668.19##	3713.86±456.52##	2401.90±240.50##	39.34±1.72##	2787.69±153.14##	2171.62±134.66##	308.04±17.51##	14.21±0.92##
MHBFC 6 mg/kg	5191.13±656.23*	3312.68±469.27	1878.44±206.92**	36.29±1.85*	2567.12±165.47*	2049.72±149.55	258.70±14.90**	12.66±0.93*
MHBFC 12 mg/kg	4418.90±815.12**	2962.43±559.09*	1456.47±259.56** $	32.99±0.81** $$	2364.39±215.87**	1935.63±180.40*	214.38±18.80** $$	11.08±0.37** $$
Lisenopril 15 mg/kg	4496.72±448.41**	3061.18±323.87*	1435.55±145.42**	31.96±1.46**	2390.94±118.50**	1972.41±104.92*	209.26±12.77**	10.62±0.64**

TAA: Area of total aorta; LA: Area of lumen; CSA: cross-sectional area; AD: aorta diameter.

## P<0.01 vs Sham group;

*P<0.05,

**P<0.01 vs Model group;

$ P<0.05,

$$ P<0.01 vs MHBFC 6 mg/kg.

### 3.3: MHBFC Improves the Left Ventricular Hypertrophy Induced by a Pressure Overload

**Figure 2:**
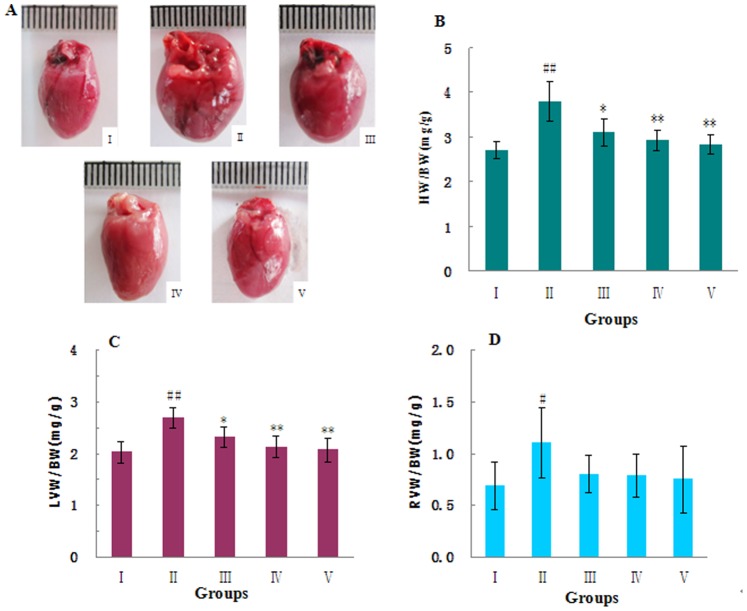
Effects of MHBFC on HW, HW/BW, LVW/BW, and RVW/BW in pressure-overload rats. (A) Representative figure of heart macroscopic image; (B–D) statistic results. I: Sham group; II: Model group; III: MHBFC 6 mg kg^−1^ group; IV: MHBFC 12 mg kg^−1^ group; V: Lisinopril 15 mg kg^−1^ group. Cardiac hypertrophy was characterized by increases in the HW/BW, LVW/BW, and RVW/BW ratios, which could be reversed by MHBFC at all doses for 6 weeks. The data are expressed as the mean±SD, n = 6.^ #^P<0.05, ^##^P<0.01 vs. Sham group; *P<0.05, **P<0.01 vs. Model group.

**Figure 3:**
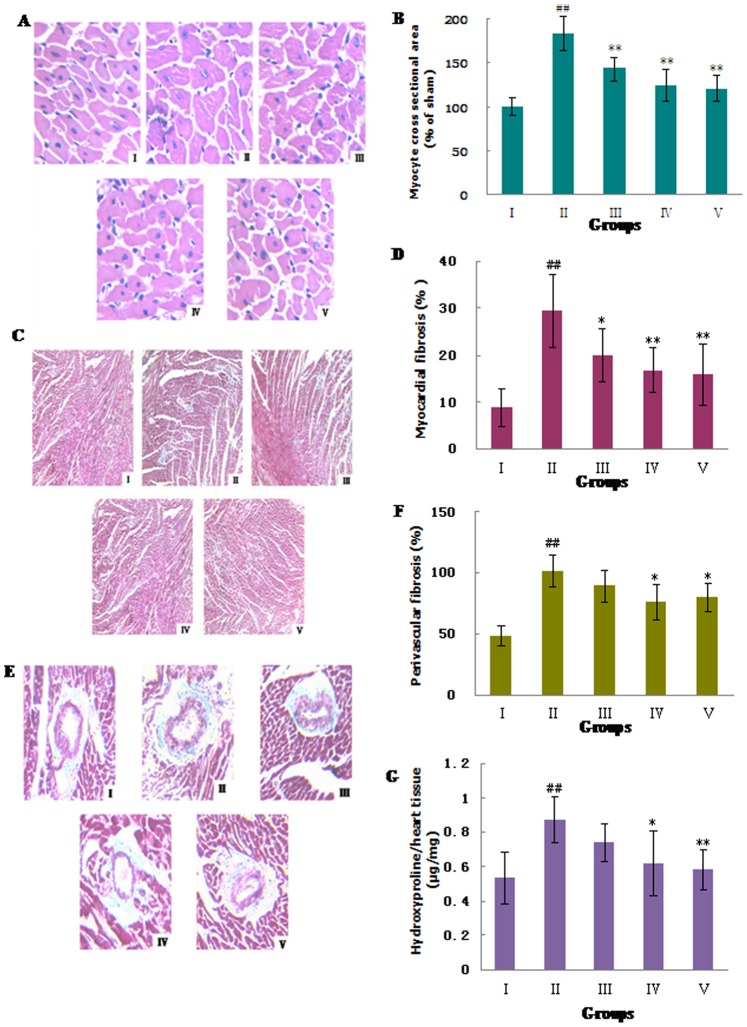
Effects of MHBFC on myocyte cross-sectional area, myocardial fibrosis, perivascular fibrosis, and hydroxyproline content in cardiac tissue of pressure-overload rats. (A) Representative figure of myocyte cross-section (HE stain, ×400); (B) statistic results of myocyte cross-section area; (C) representative figure of myocardial fibrosis (Masson’s stain, ×100); (D) statistic results of myocardial fibrosis; (E) representative figure of perivascular fibrosis (Masson’s stain, ×100); (F) statistic results of perivascular fibrosis; (G) hydroxyproline content in cardiac tissue. I: Sham group; II: Model group; III: MHBFC 6 mg kg^−1^ group; IV: MHBFC 12 mg kg^−1^ group; V: Lisinopril 15 mg kg^−1^ group. The myocyte cross-sectional area, levels of myocardial and perivascular fibrosis, and the hydroxyproline content all increased significantly when compared with the sham-operated rats. MHBFC at dose of 12 mg/kg for 6 weeks could reverse all these pathological changes in LVH parameters, and MHBFC at dose of 6 mg/kg for 6 weeks could reduced the myocyte cross-sectional area and level of myocardial fibrosis. The data are expressed as the mean±SD, n = 6.^ #^P<0.05, ^##^P<0.01 vs. Sham group; *P<0.05, **P<0.01 vs. Model group.

Results for all groups at 6 weeks after AAB are shown in Figure 2. LVH was characterized by increases in the HW/BW and LVW/BW ratios, whereas the BW showed no significant difference between groups (Table 3). The histology of the hearts from the AAB-treated rats showed that the myocyte cross-sectional area as well as the levels of myocardial and perivascular fibrosis all increased significantly compared with the sham-operated rats (Figure 3A–F). The hydroxyproline content reflected the collagen level in cardiac tissue, and the degree of myocardial fibrosis increased by 64.78% in AAB-treated rats when compared with sham-operated controls (Figure 3G). MHBFC at dose of 12 mg/kg for 6 weeks could reverse all these pathological changes in LVH parameters, and MHBFC at dose of 6 mg/kg for 6 weeks could reduce the myocyte cross-sectional area and level of myocardial fibrosis.

### 3.4: MHBFC Prevents the Progression of Hypertrophy to Cardiac Failure

**Figure 4:**
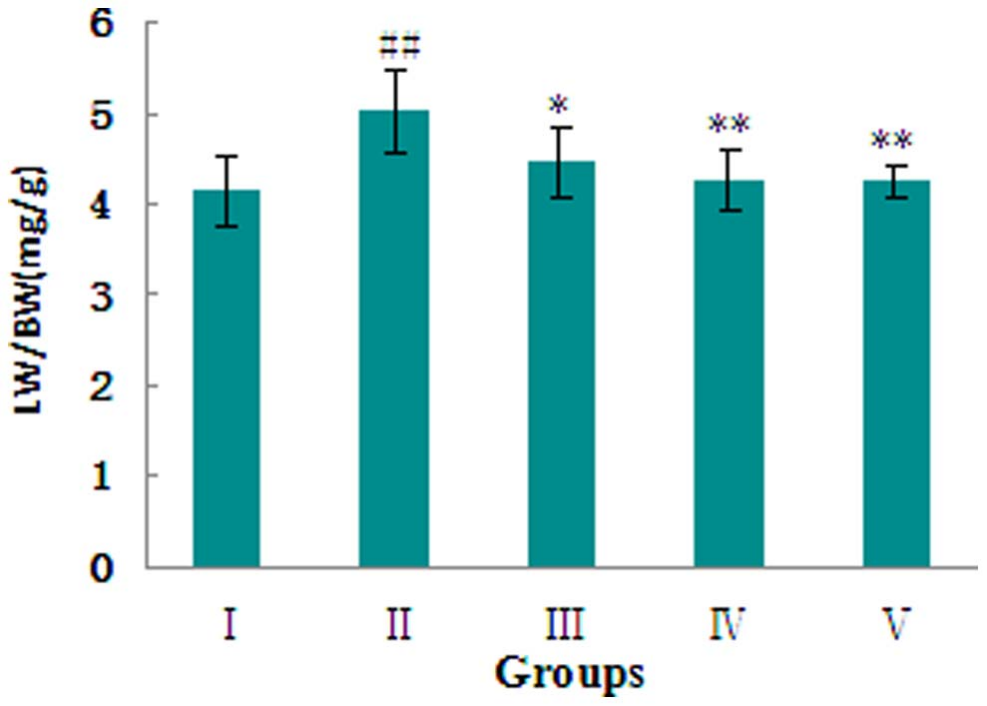
Effects of MHBFC on lung index (LW/BW) in pressure-overload rats. I: Sham group; II: Model group; III: MHBFC 6 mg kg^−1^ group; IV: MHBFC 12 mg kg^−1^ group; V: Lisinopril 15 mg kg^−1^ group. The LW/BW ratio increased when compared with sham-operated rats, which could be completely reversed by treatment with MHBFC at all doses for 6 weeks. The data are expressed as the mean±SD, n = 6.^ #^P<0.05, ^##^P<0.01 vs. Sham group; *P<0.05, **P<0.01 vs. Model group.

**Figure 5:**
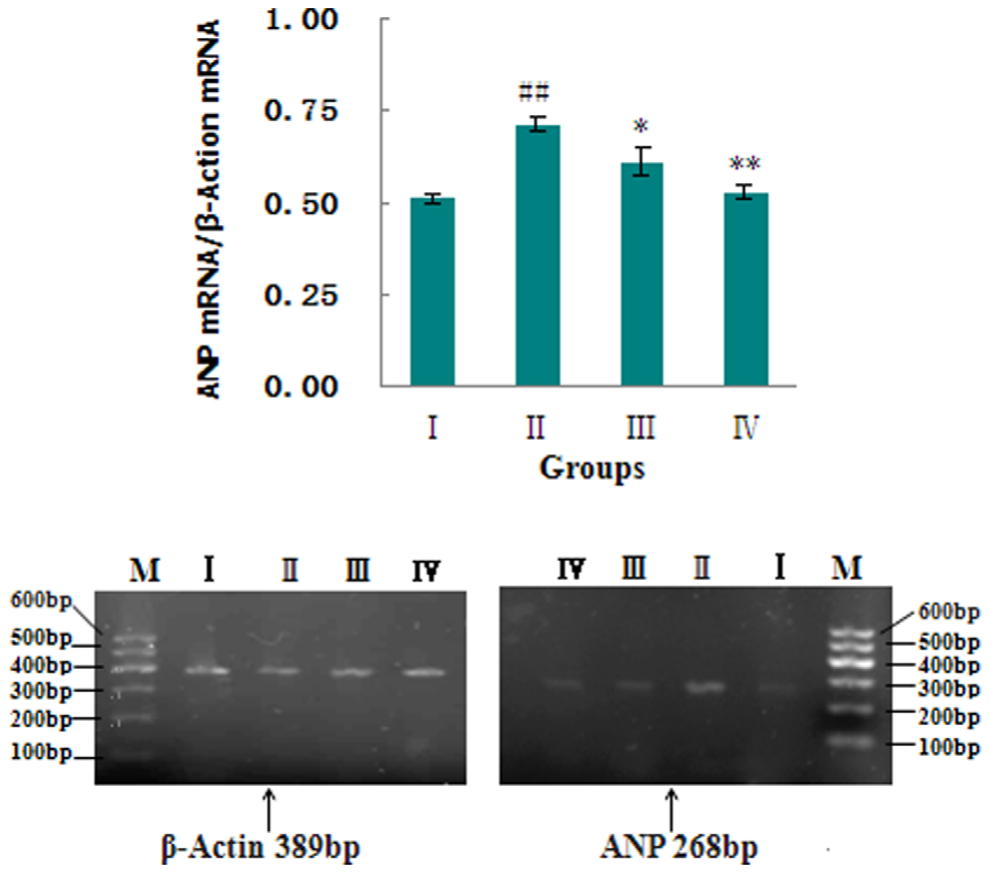
Effects of MHBFC on atrial natriuretic peptide (ANP) mRNA expression in LV tissue of pressure-overload rats. I: Sham group; II: Model group; III: MHBFC 6 mg kg^−1^ group; IV: MHBFC 12 mg kg^−1^ group. ANP is the molecular marker of heart failure. The ANP mRNAs were overexpressed compared with sham-operated rats, which could be completely reversed by treatment with MHBFC at all doses for 6 weeks. The data are expressed as the mean±SD, n = 3.^ #^P<0.05, ^##^P<0.01 vs. Sham group; *P<0.05, **P<0.01 vs. Model group.

The initiation and transition from LVH to heart failure in AAB-treated rats is characterized by right ventricular hypertrophy, pulmonary congestion and overexpression of ANP, which is a molecular marker for heart failure. [23] The RVW/BW (Figure 2D) and LW/BW (Figure 4) ratios were increased, and the ANP mRNAs (Figures 5) were overexpressed when compared with sham-operated rats. These changes could be completely reversed by treatment with MHBFC at all doses for 6 weeks.

### 3.5: Endothelial Mechanisms

A battery of tests was performed to investigate the endothelial mechanisms by which MHBFC reverses cardiac remodeling in the AAB-treated rats.

#### 3.5.1: Nitric oxide pathway

**Figure 6:**
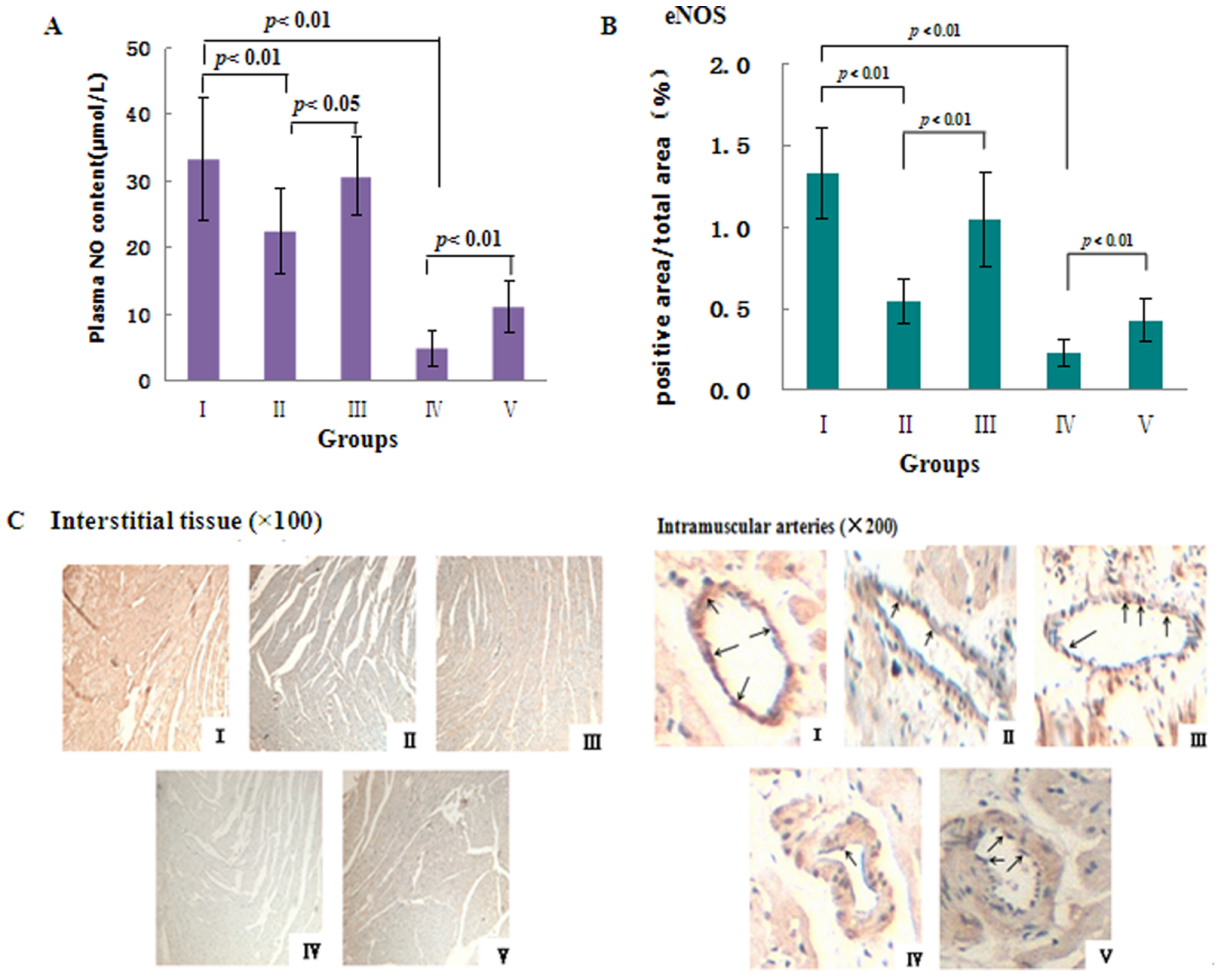
Effects of MHBFC on plasma nitric oxide content (A), endothelial nitric oxide synthase (eNOS) protein (B and C) expression in cardiac tissue of pressure-overload rats. I: Sham group; II:Model group; III: MHBFC 12 mg/kg group; IV: L-NAME 50 mg/kg; V: MHBFC 12 mg/kg +L-NAME 50 mg/kg; M, marker. (C) eNOS protein expression tested with immunohistology stain in interstitial tissue of myocardium and intramuscular arteries in hearts. Compared with the sham-operated rats, the plasma nitric oxide levels and the eNOS protein expression levels in the AAB-treated rat hearts decreased significantly, and this decrease could be prevented by treatment with MHBFC at 12 mg kg^−1^. L-NAME at 50 mg kg^−1^ could abolish these facilitatory effects of MHBFC. The data are expressed as the mean±SD, n = 3–6.^ #^P<0.05, ^##^P<0.01 vs. Sham group; *P<0.05, **P<0.01 vs. Model group.

The plasma NO levels of AAB-treated rats decreased significantly 6 weeks after AAB (P<0.01), and this decrease could be prevented by treatment with MHBFC (Figure 6A). L-NAME (50 mg kg^−1^), which is an inhibitor of NOS, could abolish these facilitatory effects of MHBFC (Figure 6A). Compared with the sham-operated rats, the eNOS protein expression levels in the AAB-treated rat hearts decreased significantly (P<0.01). MHBFC at 12 mg/kg significantly increased the eNOS protein expression levels (P<0.01 vs. model), which could also be abolished by treatment with L-NAME (Figure 6B–C). Thus, the enhanced NO signaling system, which was induced by MHBFC treatment, might be responsible for reversing the cardiac remodeling that was induced by a pressure overload.

#### 3.5.2: Endothelin pathway

**Figure 7:**
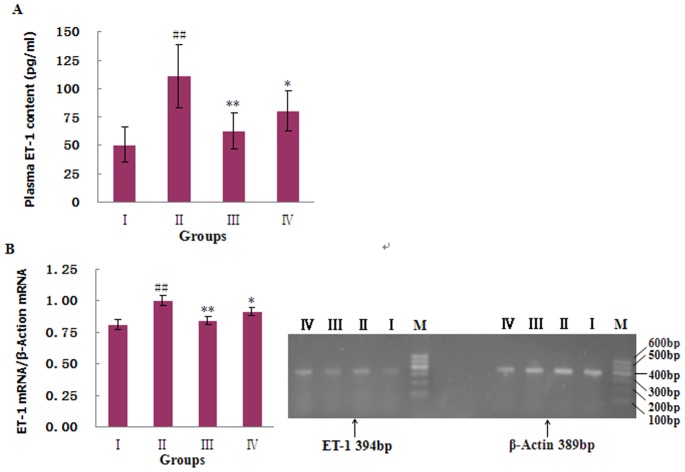
Effects of MHBFC on plasma contents of endothelin-1(ET-1) (A), and ET-1 mRNA (B) expression in cardiac tissue of pressure-overload rats. I: Sham group; II: Model group; III: MHBFC 12 mg kg^−1^ group; IV: MHBFC 6 mg kg^−1^ group; M, marker. The plasma contents and gene expression levels of ET-1 increased significantly 6 weeks after abdominal aortic banding. Treatment with MHBFC for 6 weeks at all doses significantly decreased the plasma contents and overexpression of ET-1 in cardiac tissue to nearly normal levels. The data are expressed as the mean±SD, n = 3–6.^ #^P<0.05, ^##^P<0.01 vs. Sham group; *P<0.05, **P<0.01 vs. Model group.

**Figure 8:**
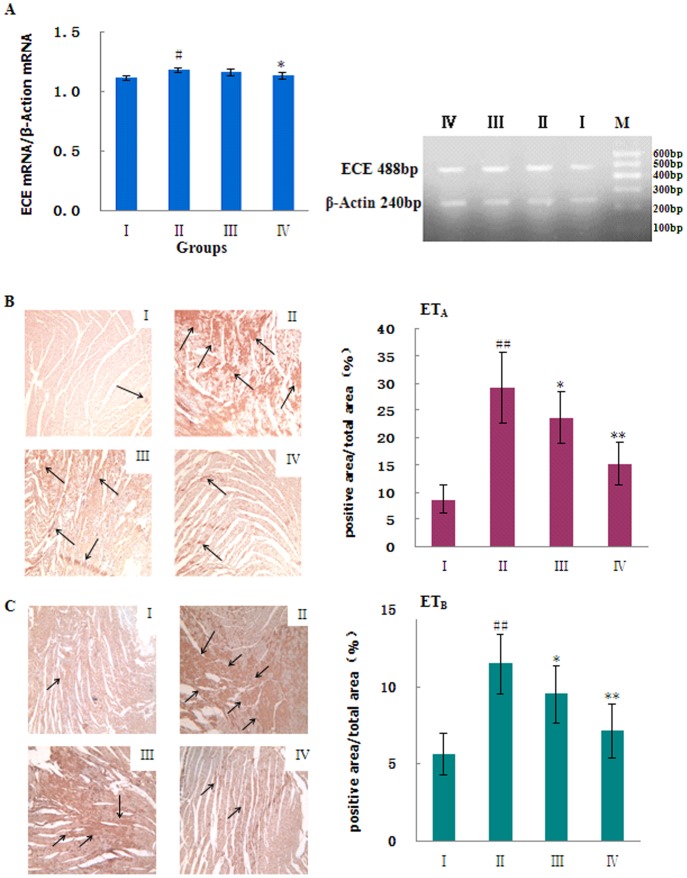
Effects of MHBFC on endothelin-converting enzyme (ECE) mRNA (A), endothelin receptor A (ET_A_) (B, immunohistology stain, ×100), and endothelin receptor B (ET_B_) (C, immunohistology stain, ×100) expression in cardiac tissue of pressure-overload rats. I: Sham group; II: Model group; III: MHBFC 6 mg kg^−1^ group; IV: MHBFC 12 mg kg^−1^ group; M, marker. The gene expression levels of ECE and the cardiac tissue protein levels of ET_A_ and ET_B_ measured by immunohistochemistry increased significantly 6 weeks after abdominal aortic banding. Treatment with MHBFC for 6 weeks significantly decreased the overexpression of ECE genes and the increased production of ET_A_ and ET_B_ proteins in cardiac tissue to nearly normal levels. The data are expressed as the mean±SD, n = 3–6.^ #^P<0.05, ^##^P<0.01 vs. Sham group; *P<0.05, **P<0.01 vs. Model group.

The plasma concentrations and the gene expression levels of ET-1 and ECE increased significantly 6 weeks after AAB (P<0.01; Figures 7 and 8A). The cardiac tissue protein levels of ET_A_ and ET_B_, as measured by immunohistochemistry, also increased significantly in the AAB-treated rats (P<0.01; Figure 8B–C). Thus, pressure overloading in the AAB-treated rats caused increased synthesis and release of ET-1, as well as increased expression levels of endothelin receptors. Treatment with MHBFC for 6 weeks significantly decreased the elevated plasma levels of ET-1, the overexpression of ET-1 and ECE genes, and the increased production of ET_A_ and ET_B_ proteins in cardiac tissue to nearly normal levels (Figures 8). Thus, MHBFC appeared to counteract the cardiac remodeling, which was induced by a pressure overload, by inhibiting the endothelin signaling system.

## Discussion

Most hypertensive animals that are used in various models of hypertension develop cardiac hypertrophy leading to heart failure, which has been characterized extensively, parallel with the rise in blood pressure. Examples of such models are various types of hypertensive rats (in particular, the spontaneously hypertensive rat) and rats, guinea pigs or ferrets that are subjected to aortic or pulmonary artery banding [24]. In this model, the abdominal aorta is banded above the renal arteries in rats to induce cardiovascular remodeling and hypertension [25]. Early hypertension arises from the activation of the renin–angiotensin system (RAS), and compensatory LVH develops, which eventually leads to heart failure. In the present experiment, decompensatory cardiac remodeling was characterized by pulmonary congestion and right ventricular hypertrophy (RVH) 6 weeks after AAB [21], [22]. This AAB rat model was characterized by LVH, LV functional disorders, pulmonary congestion, and RVH, along with hypertension.

MHBFC is a new compound that we have isolated and identified from a 60% ethanol extract of the MKL root. Our previous studies have demonstrated that extracts of MKL roots have antihypertensive, antioxidative, anti-inflammatory effects [13]–[16]. However, the possible clinical use of MHBFC for the treatment of hypertensive heart disease has not been studied. Using an aortic stenosis model, this study is the first to evaluate the improvement by MHBFC on cardiovascular remodeling induced by pressure overloading. The results suggest that MHBFC can prevent hypertension, cardiovascular remodeling, and the progression of cardiac hypertrophy to heart failure, which is induced by pressure overloading. Thus, MHBFC might be a suitable therapy for patients with hypertensive heart disease.

LVH has been recognized as an important cardiovascular risk factor. Hypertensive disease is the most frequent background of LVH, and it is generally felt that anti-hypertensive treatment should not only lower blood pressure but also cause the regression of LVH [2], [26]. That MHBFC improved LVH in our study indicates that MHBFC is beneficial against hypertensive cardiovascular events; this result is promising because an antihypertensive drug that can decrease BP effectively does not necessarily mean that it can reverse LVH. LVH is the one of the major causes of heart failure [27], [28], and because ANP plays an important role in the regulation of cardiovascular homeostasis that maintains blood pressure, ANP has emerged as a potentially important clinical biomarker of LVH [29], [30]. In the present study, the finding of pulmonary congestion, RVH, and the overexpression of ANP mRNAs indicated that cardiac functions were decompensatory in this rat model and that LVH progressed gradually to heart failure. MHBFC could effectively prevent this progression; therefore, MHBFC might be beneficial against hypertensive heart disease and congestive heart failure.

Hypertension evokes detrimental changes in the arterial vessel wall that facilitate stiffening and thus lead to a further rise in mean blood pressure, eventually causing heart failure [31]. Here, we observed hypertensive vascular remodeling of the upper thoracic aorta that was exposed to a pressure overload and systemic hypertension that was induced by narrowing the abdominal aorta; these symptoms could be reversed by treatment with MHBFC. These results suggest that MHBFC can reverse both cardiac remodeling and vascular modeling.

The dysfunction of the endothelium has been implicated in the pathophysiology of different forms of cardiovascular disease, including hypertension, coronary artery disease, chronic heart failure, peripheral artery disease etc. The pathophysiological mechanisms of endothelial dysfunction were related to a decrease in the bioavailability of NO as well as from augmented ET-1 synthesis, release, or activity [6]. Because the dysregulation of the NO and endothelin systems is important in the pathogenesis of cardiac remodeling, restoring the balance between NO and ET-1 may be an attractive therapeutic strategy for cardiac remodeling. The pathogenesis of many cardiovascular diseases is associated with reduced nitric oxide (NO) bioavailability and/or increased endothelial NO synthase (eNOS)-dependent superoxide formation. In the cardiovascular system, the signaling molecule NO, which is produced by the enzyme eNOS, has a crucial role in maintaining normal vascular function, which is mediated by its vasodilating capacity and through a variety of antiatherogenic effects [32], [33]. There is evidence demonstrating that pharmacological interventions that are designed to increase eNOS-derived NO constitute a promising therapeutic approach for the amelioration of postinfarction ventricular remodeling and heart failure [34].

Endothelin (ET-1, in particular) is regarded as an autocrine/paracrine factor in the development of cardiac hypertrophy both in vivo and in vitro [35]. The actions of ET-1 are mediated through the activation of the G-protein-coupled ET_A_ and ET_B_ receptors, which are found in a variety of cells in the cardiovascular system. Based on the biological effects that are induced by ET-1, including profound vasoconstriction, proinflammatory actions, mitogenic, proliferative, and fibrotic effects, ET-1 is implicated as an important factor in the development of cardiac hypertrophy and heart failure [36], [37]. ET-1 and the ET_A_ and ET_B_ receptors have been implicated in the pathogenesis of hypertension and in cardiac remodeling. For this reason, endothelin receptor antagonists, which are now becoming available, are being investigated as potential anti-proliferative agents [35].

In the present study, we found that MHBFC could improve the function of the NO signaling system through increasing the gene and protein expression of eNOS, resulting in augmented serum NO contents. This drug also modulated the endothelin signaling system by suppressing the synthesis and release of endothelin into the blood, which diminishes the expression of ET_A_ and ET_B_ in cardiac tissue, as well by as inhibiting the responses to endothelin. Thus, MHBFC could restore the balance between the NO and endothelin signaling systems in situations of endothelial dysfunction, resulting in endothelial protection. Furthermore, our previous studies have showed that MHBFC has potential therapeutic efficacy on the *in vitro* cardiocyte apoptosis model and *in vivo* myocardial ischemia rat model [20], Therefore, MHBFC might directly affect on the cardiomyocyte through its endothelial pathway and prevent the pressure overload-induced progression of cardiac hypertrophy to cardiac failure, but whether these effects are secondary to its effects on the vascular system have not been very clear now, and the possible mechanisms remain to be further investigated.

## Conclusions

In conclusion, the present study has shown that MHBFC offers cardiac antihypertrophic properties and helps maintain hemodynamic homeostasis. MHBFC counteracted cardiac hypertrophy and prevented the progression of cardiac hypertrophy to cardiac failure that was induced by a pressure overload. The molecular mechanism was related to its regulation of endothelial function, including the augmentation of NO release and inhibition of the ET-1 system. The further mechanisms that MHBFC interferes with the pressure overload-induced progression of cardiac hypertrophy to cardiac failure will be investigate in our further research, for example the eNOS knock-out mouse model will be used to clarify if the effects of MHBFC are eNOS dependent.
