# Correction: The Effects of 17-Methoxyl-7-Hydroxy-Benzene-Furanchalcone on the Pressure Overload-Induced Progression of Cardiac Hypertrophy to Cardiac Failure

**DOI:** 10.1371/journal.pone.0276104

**Published:** 2022-10-12

**Authors:** Jianchun Huang, XiaoJun Tang, Xingmei Liang, Qingwei Wen, Shijun Zhang, Feifei Xuan, Jie Jian, Xing Lin, Renbin Huang

During the preparation of Fig 1C in this article [1], the underlying data for panel 3CI were inadvertently used to represent the data in panel 3CIV. In the updated Fig 1 below, the correct panel has been included for Fig 3CIV. Underlying data provided for the corrected Fig 1 are in S1 File. All raw data underlying the remainder of the results reported in the article are available from the first author.

**Fig 1:**
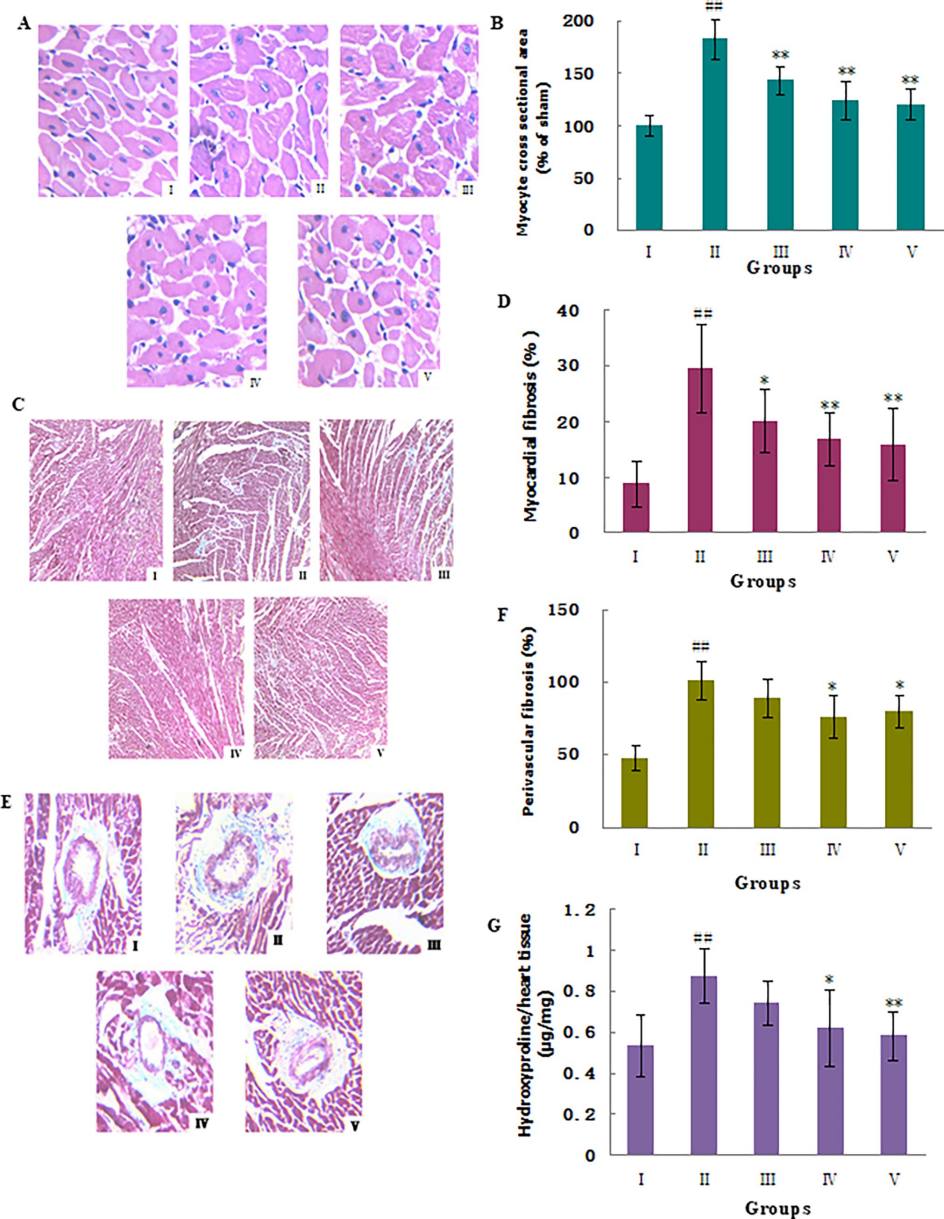
Effects of MHBFC on myocyte cross-sectional area, myocardial fibrosis, perivascular fibrosis, and hydroxyproline content in cardiac tissue of pressure-overload rats. (A) Representative figure of myocyte cross-section (HE stain, x400); (B) statistic results of myocyte cross-section area; (C) representative figure of myocardial fibrosis (Masson’s stain, x100); (D) statistic results of myocardial fibrosis; (E) representative figure of perivascular fibrosis (Masson’s stain, x100); (F) statistic results of perivascular fibrosis; (G) hydroxyproline content in cardiac tissue. I: Sham group; II: Model group; III: MHBFC 6 mg kg^-1^ group; IV: MHBFC 12 mg kg^-1^ group; V: Lisinopril 15 mg kg^-1^ group. The myocyte cross-sectional area, levels of myocardial and perivascular fibrosis, and the hydroxyproline content all increased significantly when compared with the sham-operated rats. MHBFC at dose of 12 mg/kg for 6 weeks could reverse all these pathological changes in LVH parameters, and MHBFC at dose of 6 mg/kg for 6 weeks could reduce the myocyte cross-sectional area and level of myocardial fibrosis. The data are expressed as the mean±SD, n = 6. ^#^P<0.05, ^##^P<0.01 vs. Sham group; *P<0.05, **P<0.01 vs. Model group.

## Supporting information

S1 File Underlying data supporting Fig 1A–1G. (ZIP)
