# Supplementary figures and images for: Correction: The Effects of 17-Methoxyl-7-Hydroxy-Benzene-Furanchalcone on the Pressure Overload-Induced Progression of Cardiac Hypertrophy to Cardiac Failure
Source: PLoS One. 2022 Oct 12;17(10):e0276104. doi: 10.1371/journal.pone.0276104 (PMC9555647; doi:10.1371/journal.pone.0276104)

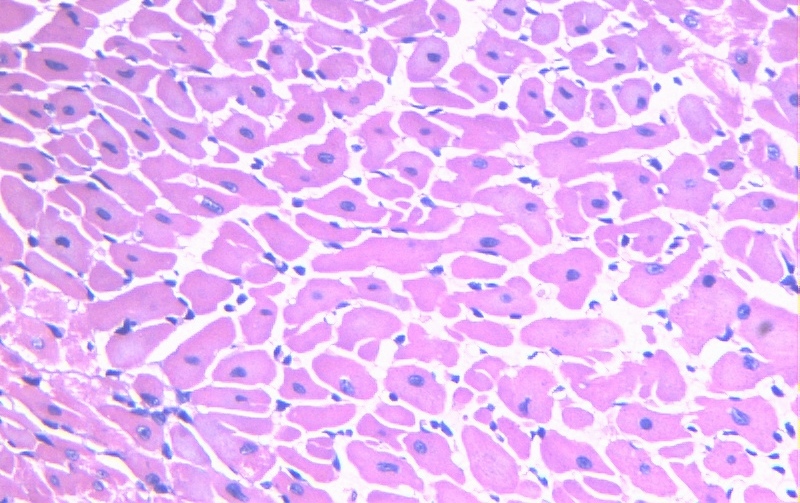

Supplement: S1 File — (ZIP) [file pone.0276104.s001.zip › correct pictures and underlying data files/the original image files for Figures 3A, C and E/Figure 3 (A) Representive figure of myocyte cross-section/I Sham group.jpg]

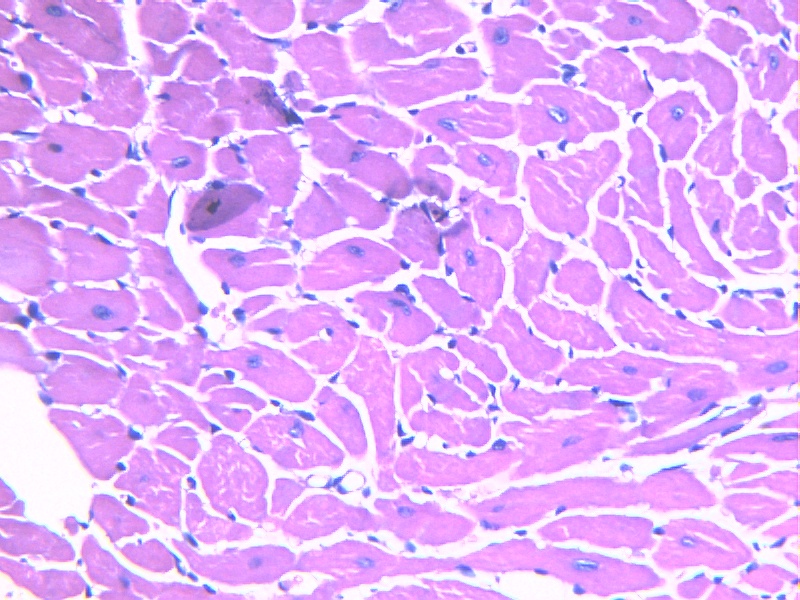

Supplement: S1 File — (ZIP) [file pone.0276104.s001.zip › correct pictures and underlying data files/the original image files for Figures 3A, C and E/Figure 3 (A) Representive figure of myocyte cross-section/II Model group.jpg]

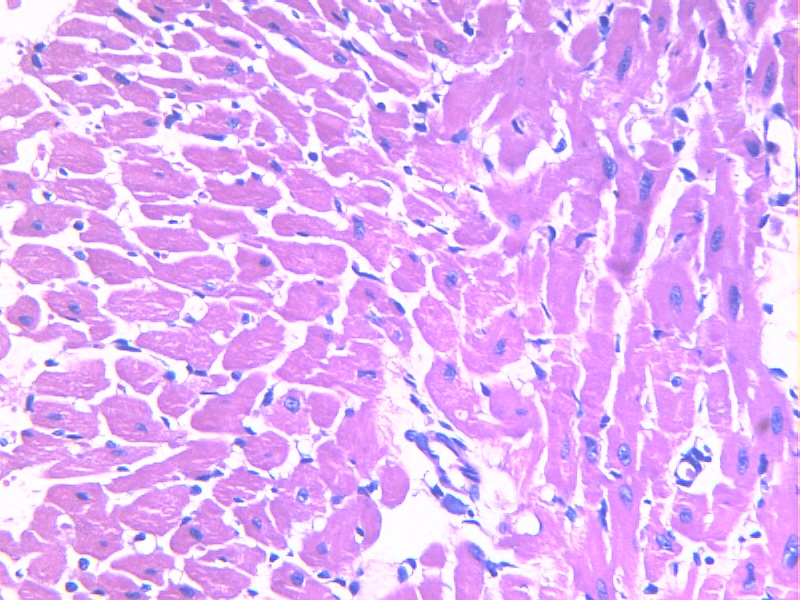

Supplement: S1 File — (ZIP) [file pone.0276104.s001.zip › correct pictures and underlying data files/the original image files for Figures 3A, C and E/Figure 3 (A) Representive figure of myocyte cross-section/III MHBFC 6 mgkg group.jpg]

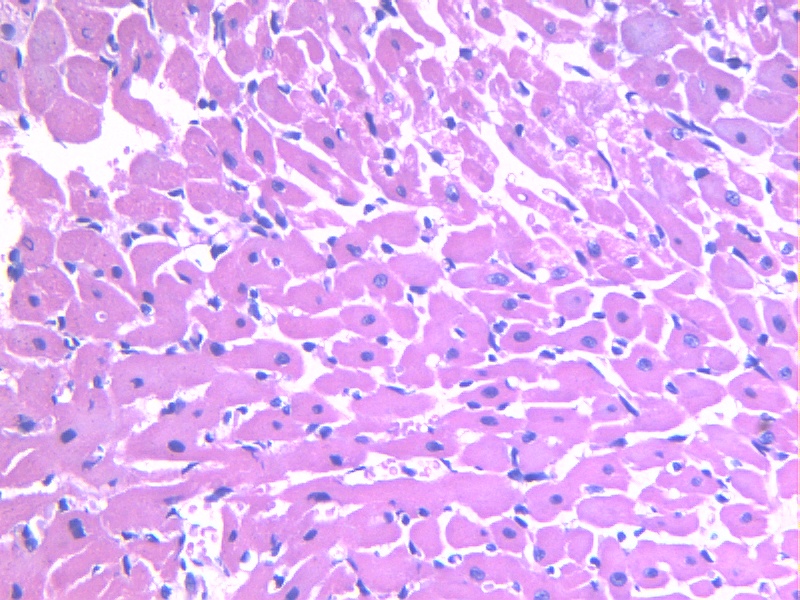

Supplement: S1 File — (ZIP) [file pone.0276104.s001.zip › correct pictures and underlying data files/the original image files for Figures 3A, C and E/Figure 3 (A) Representive figure of myocyte cross-section/IV MHBFC 12 mgkg group.jpg]

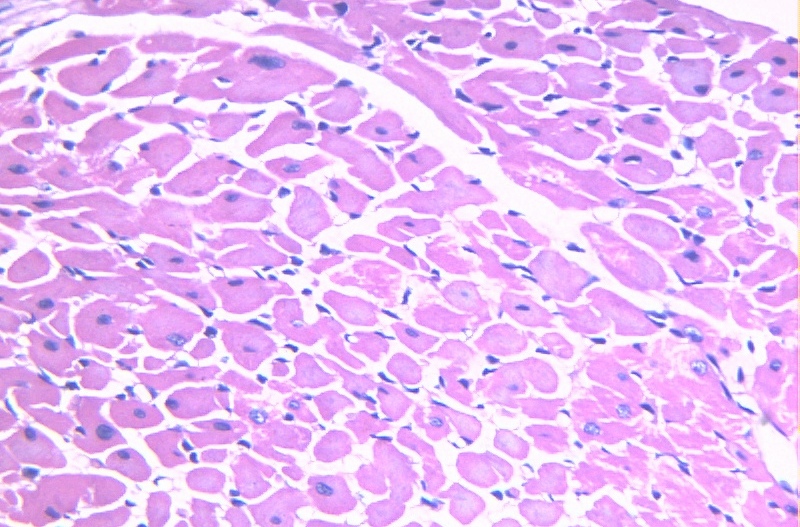

Supplement: S1 File — (ZIP) [file pone.0276104.s001.zip › correct pictures and underlying data files/the original image files for Figures 3A, C and E/Figure 3 (A) Representive figure of myocyte cross-section/V Lisenopril 15 mgkg group.jpg]

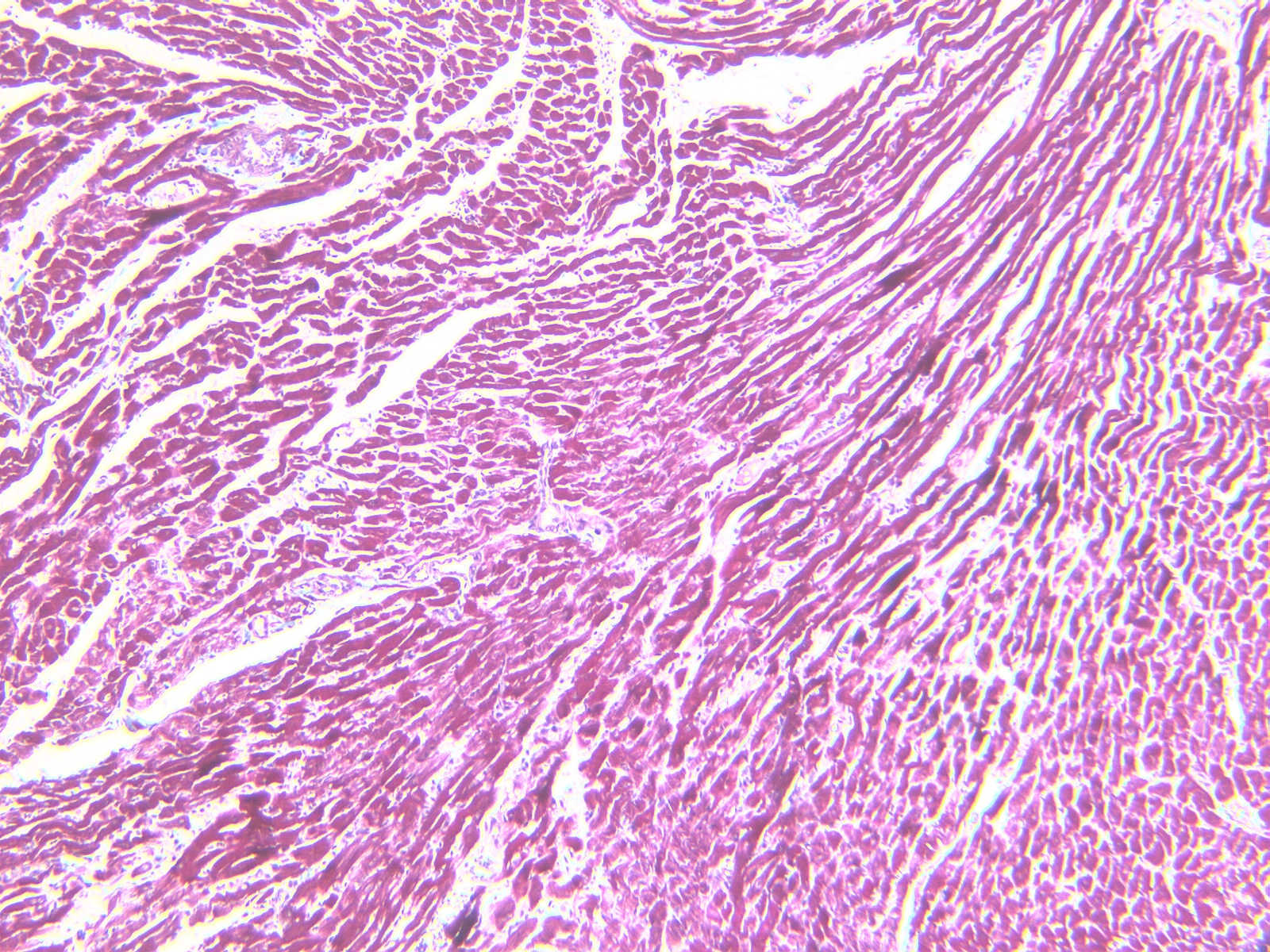

Supplement: S1 File — (ZIP) [file pone.0276104.s001.zip › correct pictures and underlying data files/the original image files for Figures 3A, C and E/Figure 3 (C) representative figure of myocardial fibrosis/I Sham group.jpg]

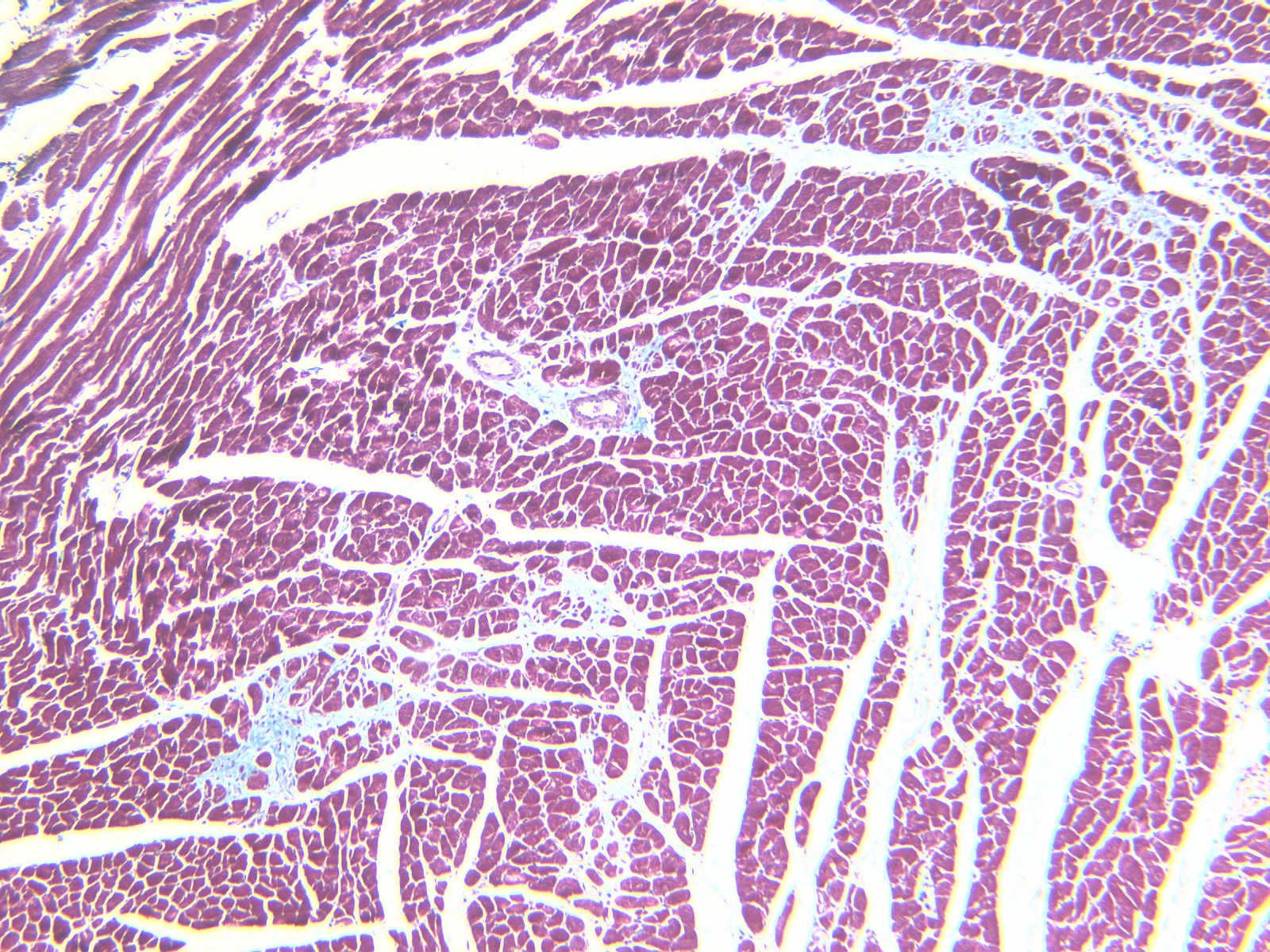

Supplement: S1 File — (ZIP) [file pone.0276104.s001.zip › correct pictures and underlying data files/the original image files for Figures 3A, C and E/Figure 3 (C) representative figure of myocardial fibrosis/II Model group.jpg]

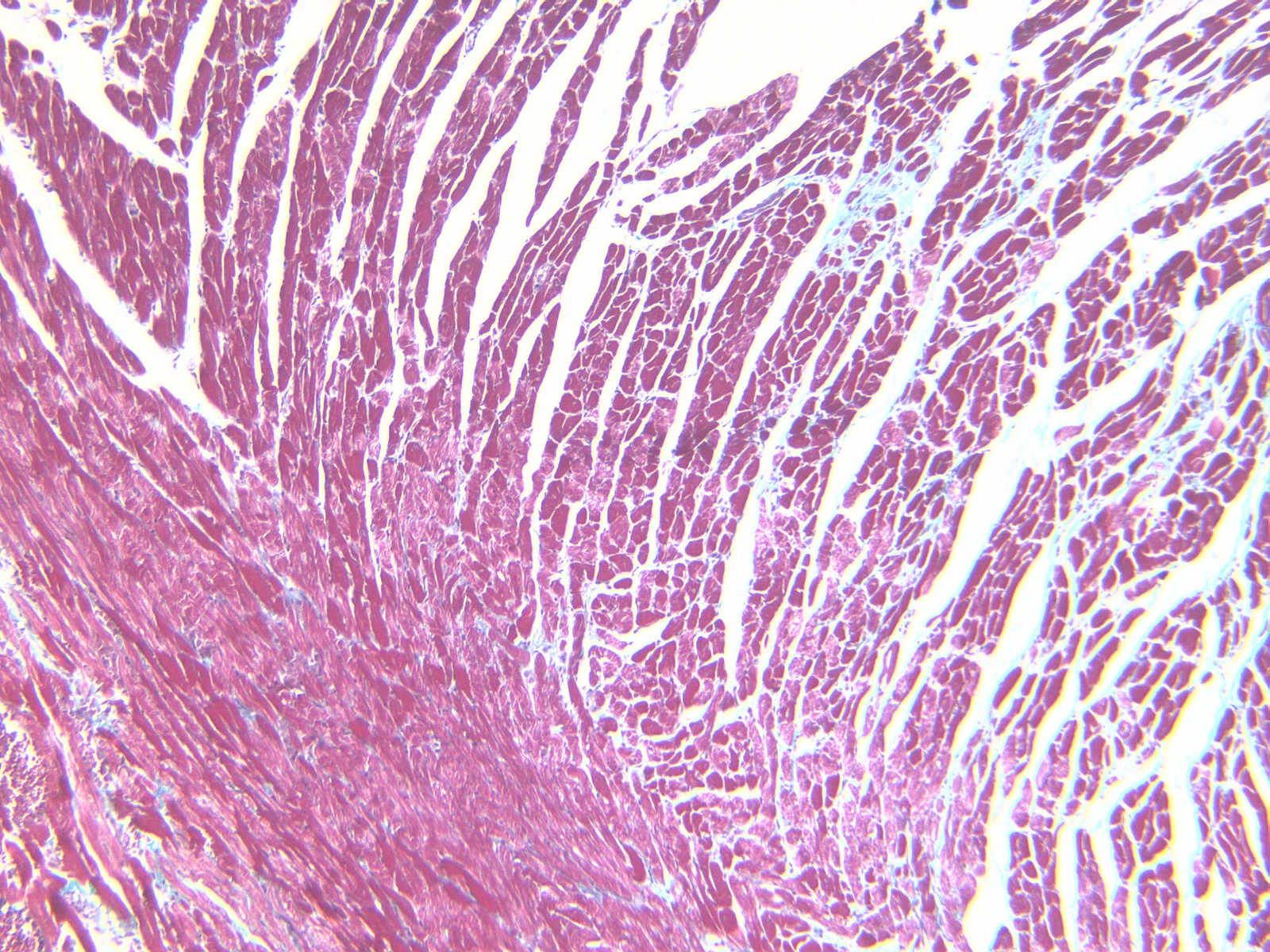

Supplement: S1 File — (ZIP) [file pone.0276104.s001.zip › correct pictures and underlying data files/the original image files for Figures 3A, C and E/Figure 3 (C) representative figure of myocardial fibrosis/III MHBFC 6 mgkg group.jpg]

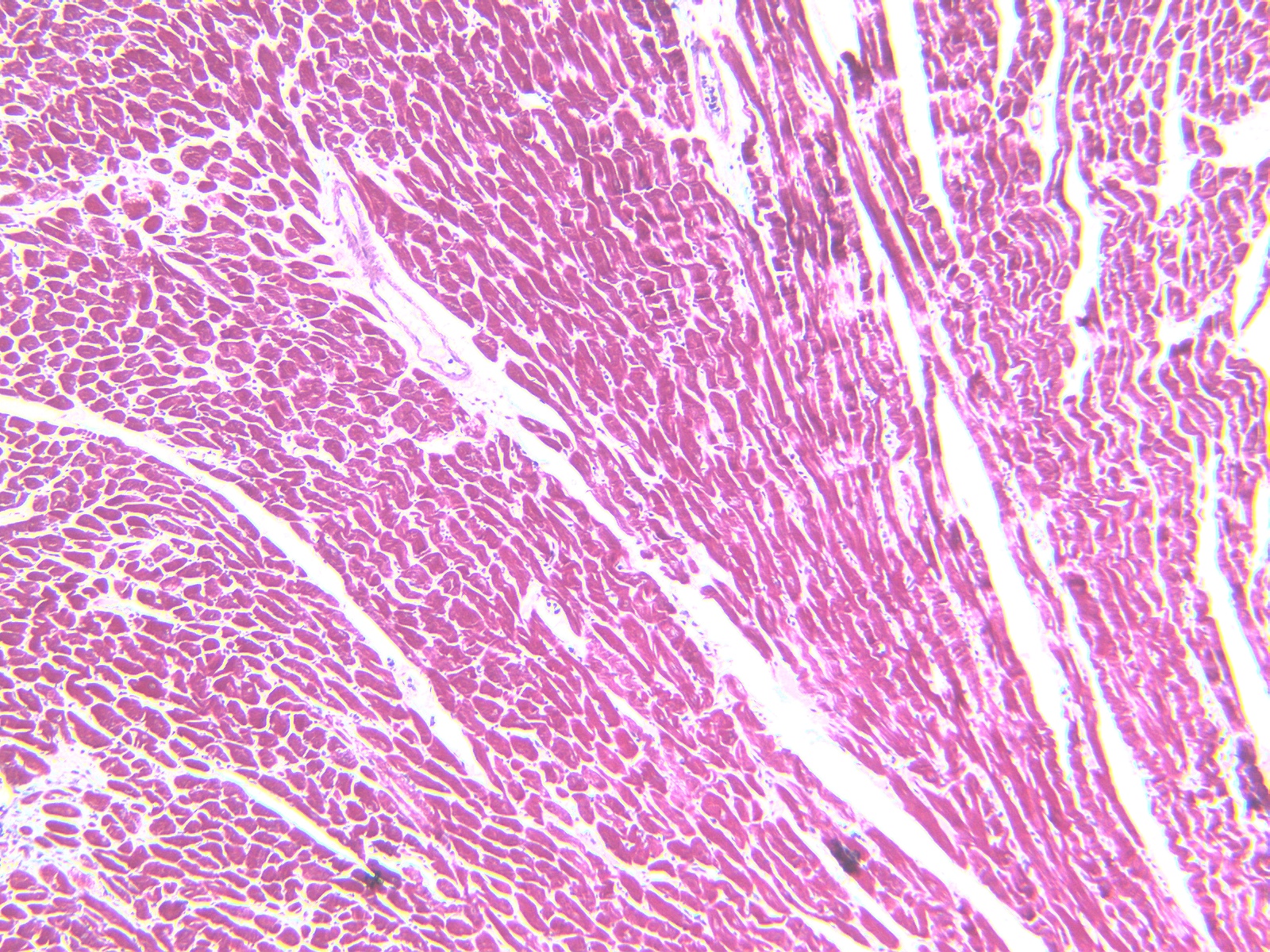

Supplement: S1 File — (ZIP) [file pone.0276104.s001.zip › correct pictures and underlying data files/the original image files for Figures 3A, C and E/Figure 3 (C) representative figure of myocardial fibrosis/IV MHBFC 12 mgkg group.jpg]

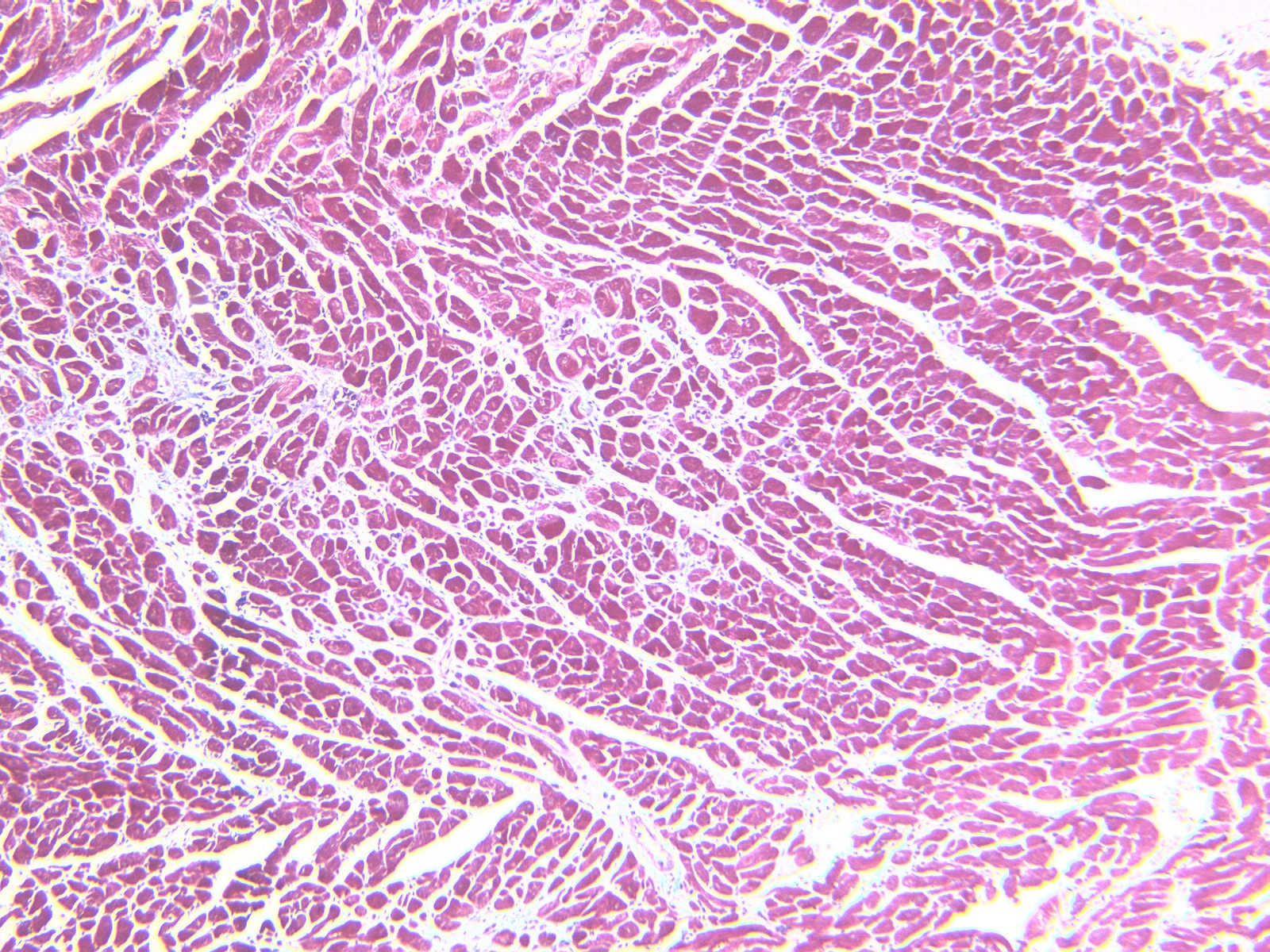

Supplement: S1 File — (ZIP) [file pone.0276104.s001.zip › correct pictures and underlying data files/the original image files for Figures 3A, C and E/Figure 3 (C) representative figure of myocardial fibrosis/VLisenopril 15 mgkg group.jpg]

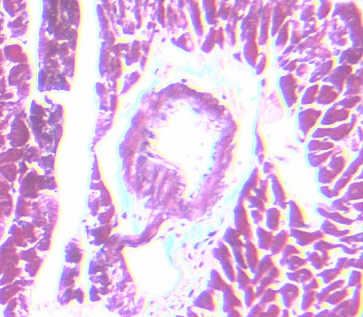

Supplement: S1 File — (ZIP) [file pone.0276104.s001.zip › correct pictures and underlying data files/the original image files for Figures 3A, C and E/Figure 3 (E) representive figure of perivascular fibrosis/I Sham group.jpg]

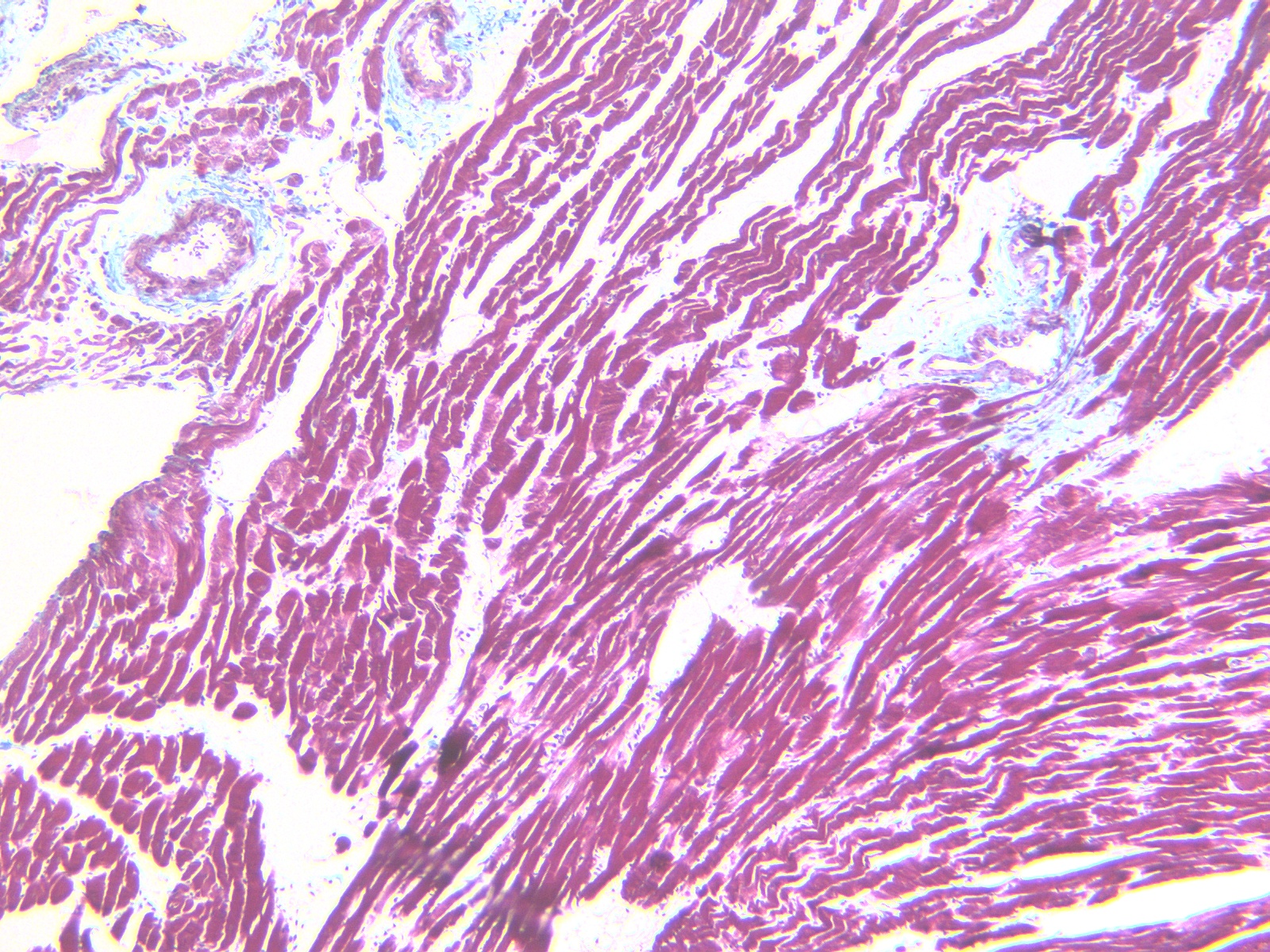

Supplement: S1 File — (ZIP) [file pone.0276104.s001.zip › correct pictures and underlying data files/the original image files for Figures 3A, C and E/Figure 3 (E) representive figure of perivascular fibrosis/II Model group.jpg]

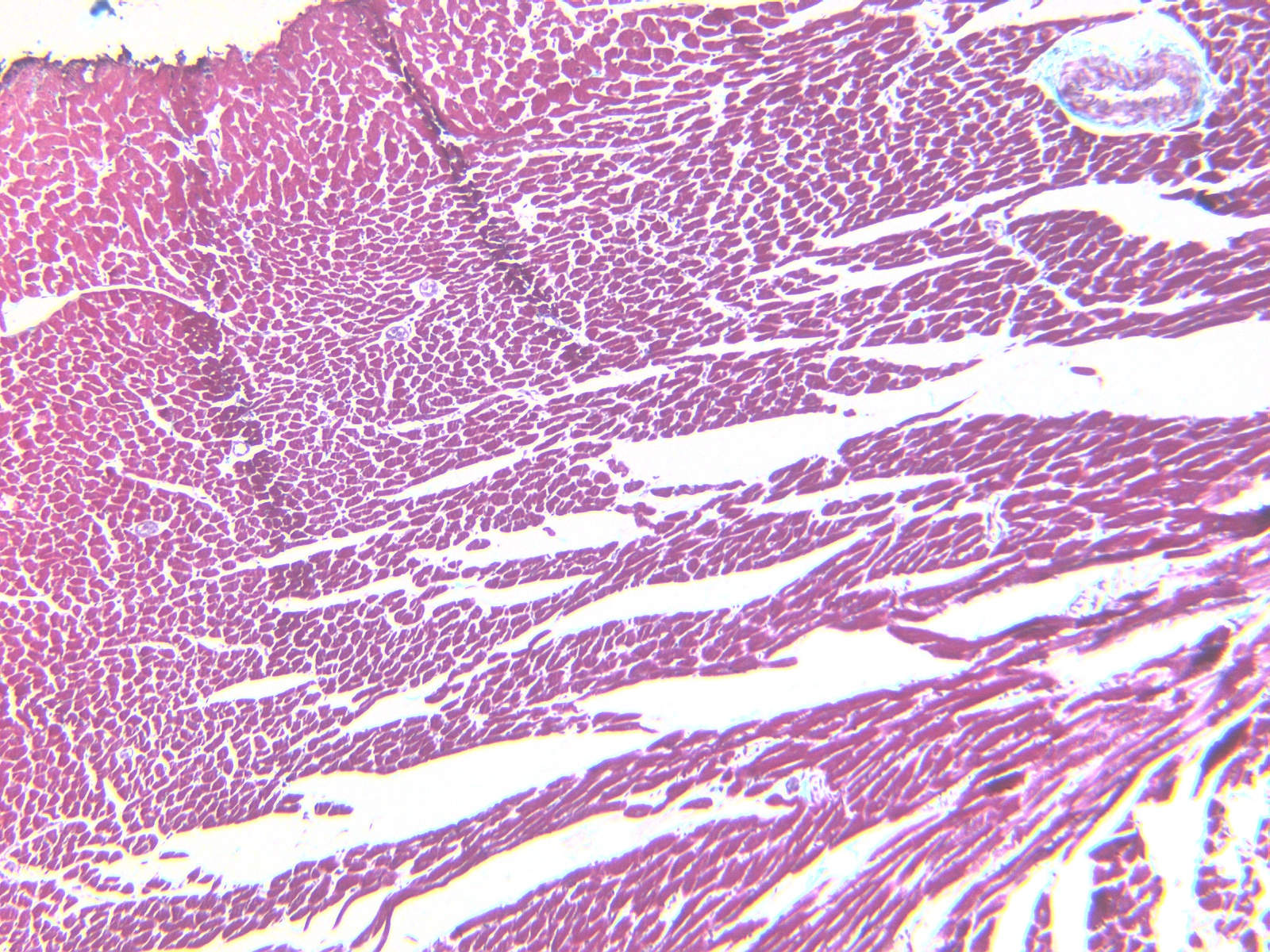

Supplement: S1 File — (ZIP) [file pone.0276104.s001.zip › correct pictures and underlying data files/the original image files for Figures 3A, C and E/Figure 3 (E) representive figure of perivascular fibrosis/III MHBFC 6 mgkg group.jpg]

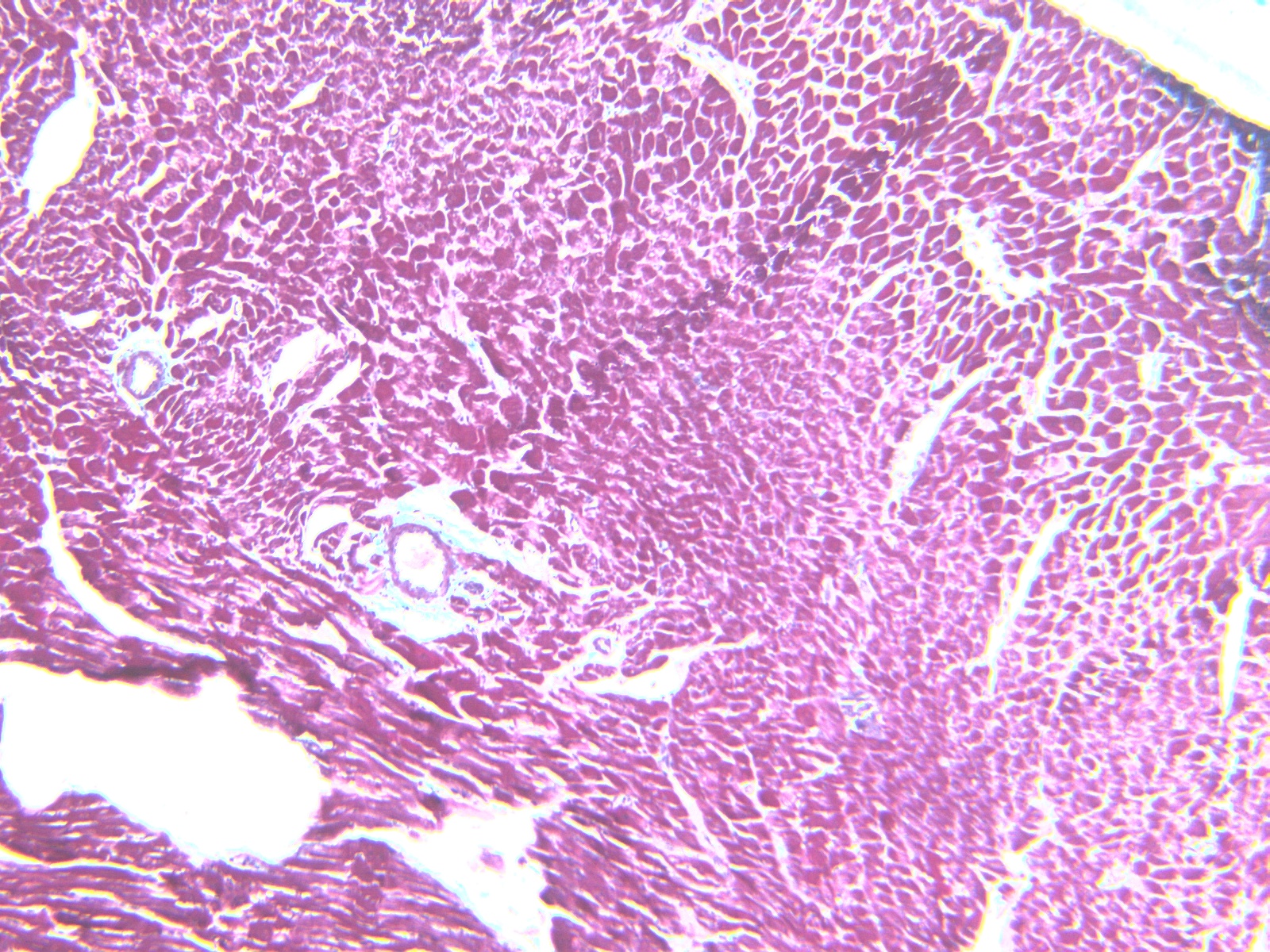

Supplement: S1 File — (ZIP) [file pone.0276104.s001.zip › correct pictures and underlying data files/the original image files for Figures 3A, C and E/Figure 3 (E) representive figure of perivascular fibrosis/IV MHBFC 12 mgkg group.jpg]

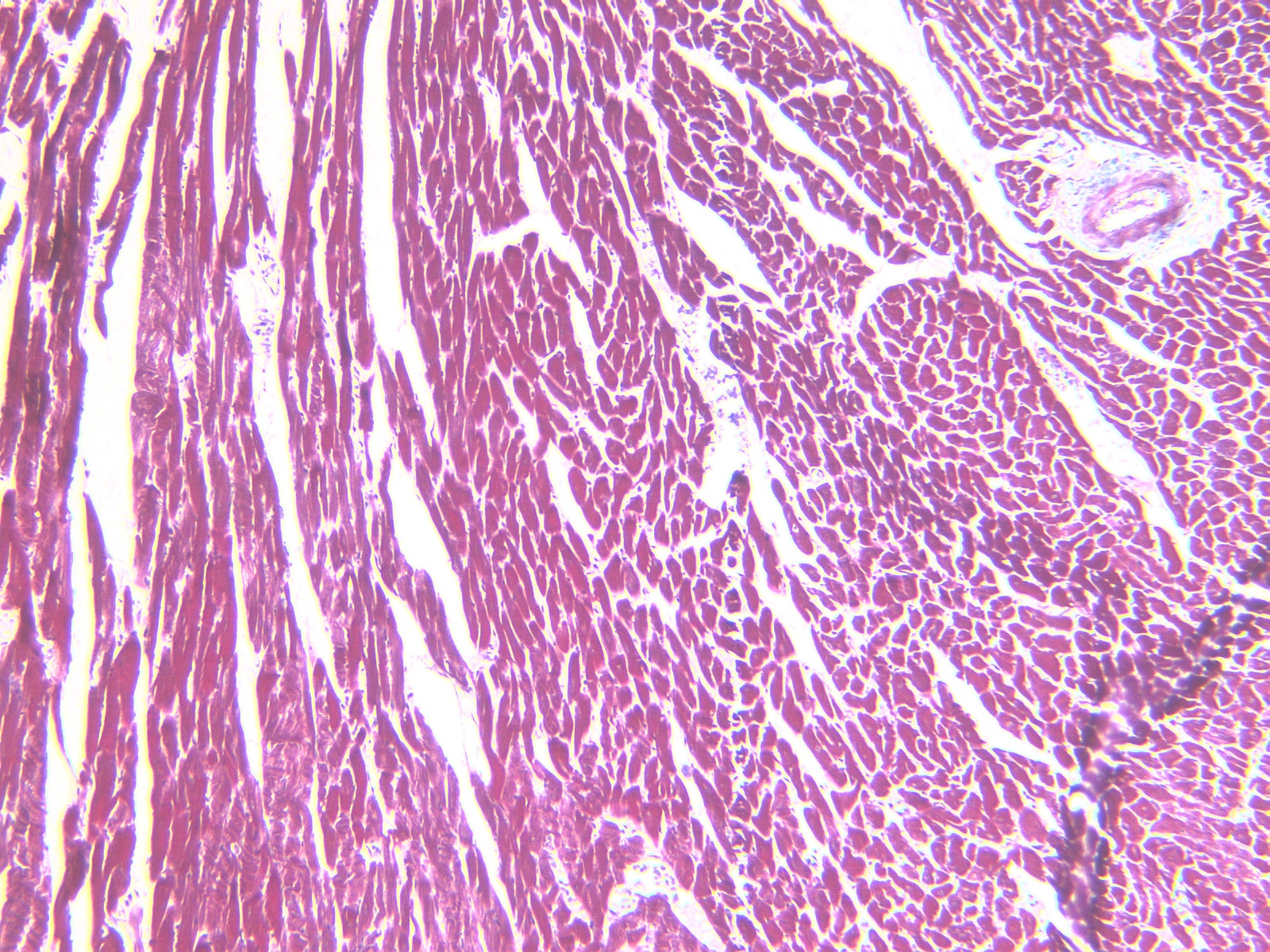

Supplement: S1 File — (ZIP) [file pone.0276104.s001.zip › correct pictures and underlying data files/the original image files for Figures 3A, C and E/Figure 3 (E) representive figure of perivascular fibrosis/V Lisenopril 15 mgkg group.jpg]
